# MRI Assessment of Lumbar Intervertebral Disc Degeneration with Lumbar Degenerative Disease Using the Pfirrmann Grading Systems

**DOI:** 10.1371/journal.pone.0048074

**Published:** 2012-12-20

**Authors:** Li-Peng Yu, Wen-Wu Qian, Guo-Yong Yin, Yong-Xin Ren, Zhi-Yi Hu

**Affiliations:** 1 Department of Spine Surgery, The First Affiliated Hospital, Nanjing Medical University, Nanjing, Jiangsu, China; 2 Department of Orthopedic Surgery, People's Hospital, Gaochun, Jiangsu, China; Charité University Medicine Berlin, Germany

## Abstract

**Background:**

To evaluate by MRI intervertebral disc degeneration in patients with lumbar degenerative disease using the Pfirrmann grading system and to determine whether Modic changes correlated with the Pfirrmann grades and modified Pfirrmann grades of disc degeneration.

**Methods:**

The clinical data of 108 surgical patients with lumbar degenerative disease were reviewed and their preoperative MR images were analyzed. Disc degeneration was evaluated using the Pfirrmann grading system. Patients were followed up and low back pain was evaluated using the visual analog scale (VAS) and the effect of back pain on the daily quality of life was assessed using Oswestry disability index (ODI).

**Results:**

Forty-four cases had normal anatomical appearance (Modic type 0) and their Pfirrmann grades were 3.77±0.480 and their modified Pfirrmann grades were of 5.81±1.006. Twenty-seven cases had Modic type I changes and their Pfirrmann grades were 4.79±0.557 and their modified Pfirrmann grades were 7.00±0.832. Thirty-six cases exhibited Modic type II changes and their Pfirrmann grades and modified Pfirrmann grades were 4.11±0.398 and 6.64±0.867, respectively. One case had Modic type III changes. Kruskal-Wallis test revealed significant difference in modified Pfirrmann grade among Modic type 0, I and II changes (*P*<0.01) but no significant difference between Modic type I and II changes (*P*>0.05). Binary regression analysis showed that Modic changes correlated most strongly with disc degeneration. Follow up studies indicated that the VAS and ODI scores were markedly improved postoperatively. However, no difference was noted in VAS and ODI scores among patients with different Modic types.

**Conclusion:**

Modic changes correlate with the Pfirrmann and modified Pfirrmann grades of disc degeneration in lumbar degenerative disease. There is no significant correlation between Modic types and surgical outcomes.

## Introduction

Magnetic resonance imaging (MRI) is the most important method for clinical assessment of intervertebral disc pathology. Losses of water content, proteoglycans and collagens that occur in degenerated intervertebral discs can be visualized on MR images with T2 weighting as hypointense signal. Radiologically, vertebral body endplate signal intensity changes on MR images have been used to diagnose degenerative disc disease and spondylosis of the lumbar spine. These signal intensity changes were first described and classified by Modic *et al.* [1] in 1988 based on a study of 474 patients and these changes have been since referred to as Modic changes. Currently, three types of Modic changes are identified. Type I changes represent bone marrow edema and inflammation and appear hypointense on T1-weighted imaging (T1WI) and hyperintense on T2-weighted imaging (T2WI). Type II changes are associated with fatty replacement of normal hemopoietic bone marrow and appear hyperintense on T1WI and isointense or slightly hyperintense on T2WI. Modic type III changes represent subchondral bone sclerosis and appear hypointense on both T1WI and T2WI. Additionally, the absence of Modic changes, indicating normal anatomical appearance, is designated as Modic type 0 by some clinical investigators [2].

Studies on clinical implications of Modic changes suggest a close association between Modic changes and the degree of disc degeneration [1], [3]. Jensen *et al.* [4] noticed an association of the extent of Modic changes with the severity of disc degeneration of adjacent lumbar discs. Kokkonen *et al.* [5] also found that endplate degeneration correlates strongly with lumbar disc degeneration and lumbar disc tear. They regard lumbar disc degeneration as an underlying cause, not a consequence, of endplate degeneration. These investigators, however, mainly studied patients with mild disc degeneration. By contrast, most surgical patients with degenerative disc disease suffer more severe disc degeneration. Currently, there is lack of studies on the relation between Modic changes and the grade of disc degeneration of surgically indicated patients. Studies are also lacking on the relation of the grades of disc degeneration among Modic type 0, I, and II changes.

The Pfirrmann grading system, as a noninvasive, simple, and convenient MR imaging method, can provide a morphologic and semi-quantitative evaluation of intervertebral disc degeneration in vivo; however, it is a subjective rating system and is inadequate for evaluating severe disc degeneration [6]. Here, we sought to investigate the relation between Modic changes and disc degeneration of severe lumbar disc degenerative disease using both the Pfirrmann grading and the modified Pfirrmann grading system and to evaluate the relation among the Pfirrmann grades and the modified Pfirrmann grades of Modic type 0, I, and II changes, and to determine whether Modic changes correlated with the grade of disc degeneration.

## Subjects and Methods

### Subjects

We retrospectively reviewed the clinical data of patients with severe degenerative lumbar disc disease at our department (Department of Spine Surgery, the First Affiliated Hospital of Nanjing Medical University, Nanjing, Jiangsu, China) between January, 2009 and October, 2011. Patients should meet all of the criteria below: a.with severe degenerative lumbar disc disease(severe lumbar disc protusion *or* lumbar spinal stenosis *or* lumbar spondylolisthesis); b. with low back pain and/or leg pain no less than six months; c. conservative treatment fails. d. had any kind of surgery below: lumbar intervertebral discectomy, laminal decompression, posterior pedicle screw fixation, intervertebral bone graft fusion, posterolateral fusion, e. with preoperative MRI data. Preoperative MRI features were analyzed as detailed below.

### MRI studies

**Figure 1:**
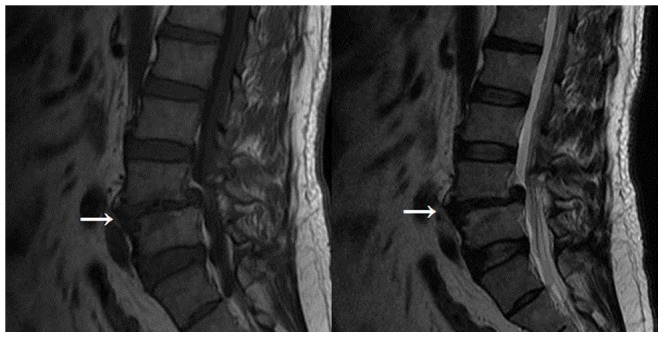
Modic type I changes (indicated by arrow) appear hypointense on. T1-weighted and hyperintense, on T 2 weighted images.

**Figure 2:**
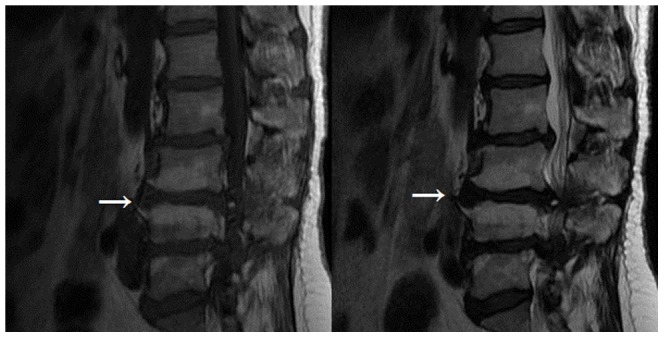
Modic type II changes (indicated by arrow) appear hyperintense on. T1-weighted and isointense or hyperintense, on T 2 weighted images.

**Figure 3:**
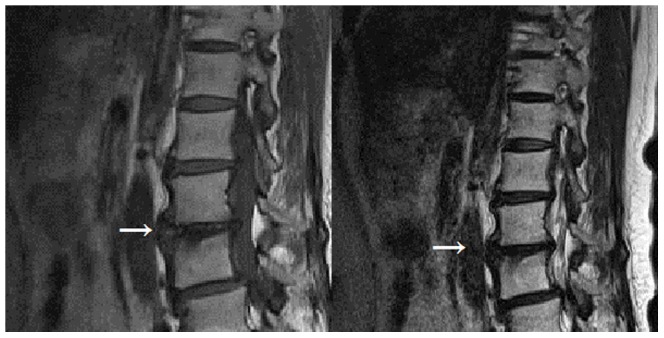
Modic type III changes (indicated by arrow) appear hypointense on. T1-weighted and hypointense, on T 2 weighted images.

Standard lumbar spine MR imaging was performed using a GE 1.5T MRI Scanner (Genesis Signa, GE Medical Systems, Milwaukee, WI). In all patients, we evaluated sagittal T1- and T2-weighted MR imaging sequences used to examine the lumbar spine. Patients examined at our facility underwent fast spin-echo sagittal T1 and T2-weighted imaging and cross-sectional T2-weighted scanning (repetition time msec/echo time msec, 3400/114; 32-cm field of view; four signals acquired; 4-mm contiguous section thickness and interslice gap 1 mm, NEX 3, variable bandwidth 31.2 kHz, and a maximum of 13 scan planes). Scanning software was used with the option of no phase wrap, variable bandwidth and no frequency wrap. Modic changes were graded as previously described [1]–[2]: type 0, normal; type I, endplate neovascularity was hyperintense on T2-weighted images and hypointense on T1- weighted images (Fig. 1); type II, endplate fatty replacement was hyperintense on T1-weighted images and isointense or hypointense on T2-weighted images (Fig. 2); type III, endplate bony sclerosis was hypointense on both T1- and T2-weighted images (Fig. 3). For variables related to Modic changes, absence or presence (type 0, 1, 2, or 3) and maximum height (craniocaudally) affected by Modic changes (only the endplate, <30% of the vertebral body, between 30% and 60% of the vertebral body, and >60% of the vertebral body) were recorded.

### Pfirrmann grading of lumbar discs

Lumbar disc grading was performed in the sagittal T2WI by two orthopedic surgeons independently who were experienced in MR imaging of the spine. They reviewed each intervertebral disc from L1–2 to L5-S1 by the Pfirrmann criteria [6], which was based on the morphological classification of intervertebral disc degeneration by Thomson *et al.* [7].The Pfirrmann grading system assesses degenerated intervertebral discs by MRI for the asymmetry in disc structure, distinction of the nucleus and the annulus, signal intensity of intervertebral discs and height of intervertebral discs and assigns grade I to V for disc degeneration. Lumbar disc degeneration was also assessed by the modified Pfirrmann grading system as described by Griffith *et al*. [8], which assigns grade 1 to 8 for disc degeneration by evaluating signal intensity of the nucleus pulposus and the inner annulus, signal intensity difference between the inner and outer part of the posterior annulus and height of intervertebral discs.

### Follow up

Patients were followed up by telephone interviews. At each follow up, low back pain was assessed using the visual analogue scale (VAS) and the effect of back pain on the daily quality of life was assessed using the Oswestry disability Index,(ODI).

### Statistical analysis

Imaging data were analyzed by two orthopedic surgeons independently who were double blind to the demographic and clinical data of patients. Independent results from the 2 reviewers were tabulated and compared. If the readers agreed on the Pfirrmann grade or the modified Pfirrmann grade for a disc, that grade was recorded for analysis. If the readers disagreed, they reviewed the images together and reached a consensus, without knowledge of the T2 measurements. Concordance between the two raters was examined using the Kappa concordance test. Group data were analyzed by the nonparametric Kruskal-Wallis test using the PASW18.0 software. Wilcoxon rank-sum test was used to compare data that did not follow a normal distribution or rank data. Correlation was analyzed by binary logistic regression. *P*<0.05 was considered statistically significant.

## Results

### Patient demographic and disease characteristics

A total of 108 cases were eligible for the study, including 43 males and 65 females and their age ranged from 38 to 79 years with a mean age of 57.8 years. They included 44 cases of spondylolisthesis, 20 cases of lumbar spinal stenosis and 44 cases of lumbar disc herniation. Lumber endplate Modic changes were present in 59.25% (64/108) of the patients, including Modic type I changes in 27 patients (25%), type II changes in 36 patients (33.33%), and type III changes in one patient (0.92). The distribution of Modic changes in spondylolisthesis, lumbar stenosis and lumbar disc herniation is shown in Table 1. Disc degeneration was observed at L2/3 (3 cases), L3/4 (4), L4/5 (36 cases), and L5/S1 (21 cases).

**Table 1:** Endplate Modic changes of degenerative lumbar disc diseases (n = 64).

	type I	type II	type III	Total
Spondylolisthesis, n(%)	15(13.89)	15(13.89)	1(0.92)	31(28.70)
Lumbar spinal stenosis, n(%)	2(1.85)	6(5.55)		8(.740)
Lumbar disc herniation, n(%)	10(9.26)	15(13.89)		25(23.15)
Total, n(%)	27(25.00)	36(33.33)	1(0.92)	64(59.25)

### Grades of disc degeneration in the study subjects

Altogether, 108 lumbar discs were analyzed and the consensus reading resulted in 14 grade III discs (12.96%), 81 grade IV discs (75.00%), and 13 grade V discs (12.03%). No grade I and II discs were observed (Table 2). In addition, these intervertebral discs were categorized by the modified Pfirrmann grading system. The consensus reading yielded 2 grade 4 discs (1.85%), 16 grade 5 discs (14.82%), 43 grade 6 discs (39,81%), 31 grade 7 discs (28.70%), and 16 grade 8 discs (14.82%). No grade 1 to 3 discs were identified (Table 3). Regarding the reproducibility of Pfirrmann grading by the two orthopedic surgeons, the intraobserver test yielded values ranging from 0.82 to 0.89, whereas the interobserver test produced values of 0.70 to 0.82. The main reason for disagreement in grading was difficulty in distinguishing the inner portion and outer annulus fibrosus. For the modified Pfirrmann grading, the intraobserver test yielded values ranging from 0.78 to 0.90, whereas the interobserver test produced values of 0.63 to 0.68. The main reason for disagreement in grading was difficulty in distinguishing the height of the discs. For the Modic changes, The Kappa score for intra- and inter-observer reliability was between 0.96 and 0.97.

**Table 2:** Pfirrmann grades of degenerative lumbar disc diseases (n = 108).

	Grade III	Grade IV	Grade V	Total
Spondylolisthesis, n(%)	2(1.85)	32(29.64)	10(9.25)	44(40.74)
Lumbar spinal stenosis, n(%)	1(0.93)	18(16.66)	1(0.93)	20(18.52)
Lumbar disc herniation, n(%)	11(10.18)	31(28.70)	2(1.85)	44(40.74)
Total, n(%)	14(12.96)	81(75.00)	13(12.03)	108(100.00)

**Table 3:** Modified Pfirrmann grades of lumbar disc degenerative diseases (n = 108).

	Grade 4	Grade 5	Grade 6	Grade 7	Grade 8	Total
Spondylolisthesis, n(%)	2(1.85)	3(2.78)	14(12.96)	15(13.89)	10(9.26)	44(40.74)
Lumbar spinal stenosis n(%)	/	5(4.63)	9(8.33)	5(4.63)	1(0.93)	20(18.52)
Lumbar disc herniation n(%)	/	8(7.41)	20(18.52)	11(10.18)	5(4.63)	44(40.74)
Total n(%)	2(1.85)	16(14.82)	43(39.81)	31(28.70)	16(14.82)	108(100.00)

### Relation between Modic changes and grades of disc degeneration

The Pfirrmann grading of Modic changes of intervertebral discs is shown in Table 4. Modic type 0 changes were found in 44 cases (40.74%) and their Pfirrmann grade was 3.77±0.48. Modic type I changes were observed in 27 cases (25.00%) and their Pfirrmann grade was 4.79±0.557. Modic type II changes were present in 36 cases (33.33%) and their Pfirrmann grade was 4.11±0.398. Modic type III changes were found in 1 patient (0.93) whose modified Pfirrmann grade was grade IV. The nonparametric Kruskal-Wallis test revealed χ^2^ = 11.453, *P* = 0.01. Modic type 0, I and II changes showed significant difference in Pfirrmann grade (*P*<0.01). The Pfirrmann grade of spondylolisthesis, lumbar spinal stenosis and disc herniation is shown in Table 4. The Pifirrmann grade of these vertebral degenerative diseases was grade III in 14 (12.96%), grade IV in 81 (75.00%, and 13 (12.03%) cases in grade V. The modified Pfirrmann grading of Modic changes of intervertebral discs is shown in Table 5. Modic type 0 changes were found in 44 cases (40.74%) and their Pfirrmann grade was 5.81±1.006. Modic type I changes were observed in 27 cases (25.00%) and their Pfirrmann grade was 7.00±0.832. Modic type II changes were present in 36 cases (36%) and their Pfirrmann grade was 6.64±0.867. Modic type III changes were found in 1 patient (0.93) whose modified Pfirrmann grade was grade 6. The nonparametric Kruskal-Wallis test revealed χ^2^ = 23.559, *P*<0.001. Modic type 0, I and II changes showed significant difference in Pfirrmann grade (*P*<0.01). Type III changes were not analyzed because of the limited sample size.

**Table 4:** Relationship between Modic changes and Pfirrmann grades of vertebral disc degenerative changes (n = 108).

	Grade III	Grade IV	Grade V	Total
Modic type 0, n(%)	11(10.18)	32(29.64)	1(0.93)	44(40.74)
Modic type I, n(%)	2(1.85)	18(16.66)	7(6.48)	27(25.00)
Modic type II, n(%)	1(0.93)	30(27.77)	5(4.63)	36(33.33)
Modic type III, n(%)		1(0.93)		1(0.93)
Total, n(%)	14(12.96)	81(75.00)	13(12.03)	108(100.00)

**Table 5:** Relationship between Modic changes and modified Pfirrmann grade of lumbar disc degeneration (n = 108).

	Grade 4	Grade 5	Grade 6	Grade 7	Grade 8	Total
Modic type 0, n(%)	2(1.85)	12(11.12)	23(21.29)	5(4.63)	2(1.85)	44(40.74)
Modic type I, n(%)			10(9.26)	8(7.40)	9(8.34)	27(25.00)
Modic type II, n(%)		4(3.70)	9(8.33)	18(16.67)	5(4.63)	36(33.33)
Modic type III, n(%)			1(0.93)			1(0.93)
Total, n(%)	2(1.85)	16(14.82)	43(39.81)	31(28.70)	16(14.82)	108(100.00)

### Relation between Pfirrmann grades of Modic changes

Wilcoxon analysis showed that the Z value of Pfirrmann grades of Modic type I and II changes was −0.358 (*P* = 0.72) and that of modified Pfirrmann grade was −1.158 (*P* = 0.249). The Z value of Pfirrmann grade of Modic type I and 0 changes was −2.618 (*P* = 0.009) and that of modified Pfirrmann grade was −4.272 (*P*<0.001). The Z value of Pfirrmann grade of Modic type II and 0 changes was −3.07 (*P* = 0.002) and that of modified Pfirrmann grade was −3.779 (*P*<0.001). Type III changes were not analyzed because of the limited sample size.

### Relation between Modic changes and grades of disc degeneration

Forty-four of our study subjects had spondylolisthesis. Thirteen (29.54%) of them had Modic type 0 changes, 15 (34.09%) Modic type I changes, 15 (34.09%) Modic type II changes, and 1 (2.27%) Modic type III changes. The relation between Modic changes and Pfirrmann grades of disc degeneration in spondylolisthesis patients is shown in Table 6. The nonparametric Kruskal-Wallis test revealed χ^2^ = 5.53 and *P* = 0.148, indicating that there was no statistical difference in Pfirrmann grades of disc degeneration in spondylolisthesis patients with either Modic type 0, I or II changes (*P*>0.05).

**Table 6:** Relationship between Modic changes and Pfirrmann grade in patients with spondylolisthesis (n = 44).

	Grade III	Grade IV	Grade V	Total
Modic type 0, n(%)	1(2.27)	12(27.27)		13(29.54)
Modic type I, n(%)		9(20.45)	6(13.64)	15(34.09)
Modic type II, n(%)	1(2.27)	10(22.73)	4(9.09)	15(34.09)
Modic type III, n(%)		1(2.27)		1(2.27)
Total, n(%)	2(4.54)	32(72.73)	10(22.73)	44(100.00)

We additionally analyzed the relation between Modic changes and modified Pfirrmann grades of disc degeneration in spondylolisthesis patients. The nonparametric Kruskal-Wallis test revealed χ^2^ = 9.63 and *P* = 0.022. Modic type 0, I and II changes showed significant difference in modified Pfirrmann grade of spondylolisthesis patients (*P*<0.05).

Forty-four of our study subjects had lumbar disc herniation. Nineteen (43.18%) of them had Modic type 0 changes, 10 (22.72%) Modic type I changes, 15 (34.10%) Modic type II changes, and no patient had Modic type III changes. The relation between Modic changes and Pfirrmann grades of disc degeneration in lumbar disc herniation patients is shown in Table 7.The nonparametric Kruskal-Wallis test revealed χ^2^ = 3.92 and *P* = 0.140, indicating that there was no statistically significant difference in the Pfirrmann grades of disc degeneration in lumbar disc herniation patients with either Modic type 0, I or II changes (*P*>0.05). The nonparametric Kruskal-Wallis test revealed χ^2^ = 7.07 and *P* = 0.029, indicating that there was statistically significant difference in modified Pfirrmann grades of disc degeneration in lumbar disc herniation patients with either Modic type 0, I or II changes (*P*<0.05).

**Table 7:** Relationship between Modic changes and Pfirrmann grade in patients with disc herniation (n = 44).

	Grade III	Grade IV	Grade V	Total
Modic type 0, n(%)	8(18.19)	10(22.72)	1(2.27)	19(43.18)
Modic type I, n(%)	2(4.54)	7(15.90)	1(2.27)	10(22.72)
Modic type II, n(%)	1(2.27)	14(31.81)		15(34.10)
Modic type III, n(%)				
Total, n(%)	11(25.00)	31(70.46)	2(4.54)	44(100.00)

Lumbar spinal canal stenosis patients were not analyzed because of the limited sample size.

### Correlation of Modic changes with disc degeneration

We further analyzed the correlation among Modic changes, disease type and disc generation by binary logistic regression. The regression yielded the following equation: M = −0.091K+1.514P−5.395, where M represents Modic changes, K represents disease type and P represents disc degeneration (F = 12.514, *P* = 0.005), (EXP: K = 0.913, P = 5.545). We found that Modic changes had the highest correlation with disc degeneration and the weakest correlation with disease type.

### Follow up results

Totally, 57 patients were followed up and the mean duration of follow up was 29.7 months (range, 10 to 36 months). They included 21 cases of Modic 0 changes, 15 cases of Modic I changes and 21 cases of Modic II changes. There was no difference in age, sex and course of illness among patients with different Modic types. At week 1 following operation and the final follow up, the VAS and ODI scores were markedly lower than preoperative VAS and ODI scores, respectively (*P*<0.01). These findings indicated that the surgical treatment could markedly reduce pain and improve functional performance of patients with lumbar disk degenerative diseases. There was no significant differences in VAS and ODI scores preoperatively, 1 week following operation and at the final follow up among patients with Modic 0, 1 or 2 changes (*P*>0.05), suggesting that there was no difference in surgical outcome among patients with different Modic types (Table 8).

**Table 8:** Preoperative and postoperative VAS and ODI scores of patients with different Modic types.

	VAS score (x±S)			ODI score (x±S)		
Modic type (n)	Preoperative	week 1 postoperative	Final follow up	Preoperative	week 1 postoperative	final follow up
type 0 (21)	6.14±1.96	1.43±1.43	0.67±1.39	0.64±0.22	0.23±0.19	0.13±0.14
type I (15)	6.33±1.95	1.67±1.11	0.73±1.28	0.57±0.25	0.17±0.14	0.09±0.14
type II (21)	6.10±2.93	1.76±1.95	1.04±1.66	0.61±0.26	0.20±0.17	0.14±0.16

## Discussion

In 1987, de Roos *et al.* [9] detected signal intensity changes at adjacent endplates in patients with degenerative lumbar disc disease. These changes were further characterized in 1988 by Modic *et al.* [1], [3] who delineated signal intensity changes in endplates in lumbar disc in 474 patients; most of them had chronic low back pain. These authors described two types of endplate and marrow changes, type I and type II [1] and subsequently added type III changes [3]. Jensen *et al.* [4] followed up 344 subjects in the general population for 4 years and found that the extent of Modic changes was associated with the severity of disc degeneration of adjacent lumbar discs. Kokkonen *et al*. [5] also found that endplate degeneration correlates strongly with lumbar disc degeneration and lumbar disc tear. However, there have been few studies on the relation between Modic changes and the grade of lumbar disc degeneration. In the current study, we evaluated endplate signal intensity changes in patients with severe degenerative lumbar disc disease and investigated the relation between Modic changes and the grades of disc degeneration. We found that approximately 60% of our patients showed abnormalities in endplate appearances in MRI; one fourth of them had Modic type I changes and one third had Modic type II changes. Analysis of the Pfirrmann grading of their lumbar discs revealed grade III (12.96%), IV (75%), or V discs (12.03%) and no grade I and II discs. Their modified Pfirrmann grading yielded grade 4 (1.85%), 5 (14.82%), 6 (39.81%), 7 (29.63%), and 8 discs (13.89%) and no grade 1 to 3 discs. These findings suggest that our subjects had more severe lumbar disc degeneration compared those reported by others [2], [4], [5]. Our Kruskal-Wallis test further revealed significant difference in the Pfirrmann grades and modified Pfirrmann grades of Modic type 0, I and II changes.

Toyone *et al*. [10] found that the incidence of Modic changes is 19% to 59% of patients with degenerative lumbar disc disease and type 1 and II changes are more commonly seen while type III changes and the mixed types are less frequently seen. The initial study by Modic *et al.* showed [1], [3] that the incidence of type II changes could reach 90%, while the study by Jensen *et al*. [4] found that the rate of new vertebral endplate signal intensity changes was 19.5%, and 84% of them belonged to Modic type I changes. The subjects in the current study were indicated for surgical treatment and had more severe degenerative lumbar disc disease and the rate of Modic changes was approximately 60%, which is consistent with that reported by other investigators [11]–[13]. Close to 60% of our patients had Modic type I and II changes and the number of patients with Modic type II changes was higher than that of patients with Modic type I changes, which is different from the finding by Jensen *et al.* [4] This could be attributed to the fact that the population in Jensen *et al*.'s study consisted of normal subjects and patients with mild lumbar disc degeneration, hence a higher number of patients with Modic type I changes. We also found in our current series that Modic changes occurred most frequently at L4/5 and L5/S1, which is consistent with those reported by others [14], [15].

Modic *et al.* initially studied 3 Modic I cases and 3 Modic II cases and they found that Modic 1 changes were associated with disruption in endplates and formation of connective tissues and blood vessles while Modic II changes were associated with fatty degeneration of the red bone marrow, which was eventually replaced by yellow marrow [1]. They believed that Modic I changes corresponded the inflammatory stage of lumbar disk degenerative diseases with the degenerating process ongoing while Modic II represented the s and prolonged fatty degeneration process in lumbar disk degenerative diseases and Modic III represented the sclerosing process in lumbar disk degenerative diseases. Burke *et al.* detected the presence of inflammatory cytokines such as interleukin 6 and 8 in lumbar disk with Modic I changes and believed that these inflammatory cytokines could be a major contributor of low back pain [16]. Ohtori *et al.* found that endplates with Modic I changes had higher levels of PGP 9.5 and TNF than normal endplates and they claimed that the growth of inflammatory cells and nerves in endplates was an important cause of low back pain [17]. Vital *et al.* also believed that Modic I changes corresponded to cartilaginous edema beneath the endplate, reflecting microscopic fractures of the cancellous bone and destruction of endplates with concomitant increase in the number of blood vessles and nerve ending and increased levels of inflammatory mediators [18].

Emch *et al.* [19] attribute Modic changes to the presence of physical pressure in the intervertebral space, nutritional factors and genetic factors. Crockt [20] formulated the internal disc disruption hypothesis, which holds that Modic changes develop as a consequence of local inflammation in the endplate resultant from persistent lumbar disc injuries. Kokkonen *et al*. [5] found that endplate changes correlated with lumbar disc degeneration. We also found here that Modic changes were associated with the grade of disc degeneration. Lumbar spinal segmental instability is an important pathological process in degenerative lumbar disc disease. Disc degeneration can cause spinal segmental instability, which in turn aggravates segmental instability, resulting in a vicious cycle. Tonyone *et al*. [10] found that patients with Modic type I changes are more prone to have spinal segmental instability than those with type II changes. It remains unclear whether spinal segmental instability causes Modic changes by aggravating disc degeneration.

The Pfirrmann grading system does not take age into full consideration and there is also interobserver discordance on Pfirrmann grade II and III of disc degeneration. In addition, there is large variation in vertebral heights for Pfirrmann grade III and IV [5]. The modified Pfirrmann grading system further divides Pfirrmann grade III and IV and categorizes disc degenerative changes into 8 grades and has improved the evaluation of disc degeneration of severe degenerative disc disease in elderly patients [8]. The limitations of the modified Pfirrmann grading system are its subjectivity in assigning grade of disc degeneration and too narrow definition of vertebral heights. In this study, we used both the Pfirrmann grading system and the modified Pfirrmann grading system to evaluate lumbar disc degeneration to overcome the deficiencies in each scoring system and further analyzed the relation between Modic changes and the Pfirrmann grade and the modified Pfirrmann grade of disc degeneration. We found that there was a significant difference in the Pfirrmann grade and the modified Pfirrmann grade among Modic type 0, I and II changes. We also showed that there was a significant difference in the modified Pfirrmann grade of disc degeneration in spondylolisthesis patients with Modic type 0, I and II changes while no difference was noted in the Pfirrmann grade. The discrepancy between the Pfirrmann grade and the modified Pfirrmann grade may be due to the possibility that the Pfirrmann grade indeed is not associated with Modic changes in spondylolisthesis Patients or the Pfirrmann grading system is insensitive to differences in disc degeneration.

Esposito *et al.* carried out a prospective study of 60 patients with low back pain who received interbody fusion surgery and they found that patients with Modic I changes showed a marked improvement in pain reduction and functional performance during 14 months of follow up while no such changes were observed in patients with Modic II changes [21]. Rikke et al. reviewed the literature on the relation between surgical outcomes and Modic changes in patients receiving various types of treatment. They found that, in paitents receiving lumbar disk replacement or functional exercise, Modic types did not impact on the prognosis of these patients [22]. Djurasovic et al. followed up 51 patients with lumbar disk disease who received interbody fusion surgery for 2 years and found that Modic changes did not correlate with the prognosis of these patients [23], which are consistent with our findings. The relation between Modic types and fusion rate still remains unclear and requires further study.

In conclusion, our current study demonstrates that Modic changes are present in 60% of our patients with severe degenerative disc disease and correlate closely with disc degeneration. Because of the retrospective nature of the current study, it remains unknown whether assessment of Modic changes and evaluation of Pfirrmann grades could detect Modic changes and disc degeneration early in the course of disease, which may lead to early intervention for forestalling the progression of disc degeneration. Further studies are needed to find a more sensitive and robust MRI scoring system for quantitatively detecting the early stage changes in degenerative vertebral disc diseases.
